# Trapezium Fracture Associated with Thumb Carpometacarpal Joint Dislocation: A Report of Three Cases and Literature Review

**DOI:** 10.1155/2018/2408708

**Published:** 2018-02-21

**Authors:** Sho Kohyama, Toshikazu Tanaka, Akira Ikumi, Yasukazu Totoki, Kosuke Okuno, Naoyuki Ochiai

**Affiliations:** Department of Orthopedic Surgery, Kikkoman General Hospital, Noda, Japan

## Abstract

Isolated trapezium fracture in combination with thumb carpometacarpal (CMC) joint dislocation is extremely rare, and no treatment consensus exists. Herein, we report 3 successfully treated cases of isolated trapezium fracture with thumb CMC joint dislocation. While good short-term results have been reported in the literature, the possibility of substantial ligament injuries that can lead to future instability of the thumb CMC joint must be noted. In order to obtain an excellent long-term clinical result, we propose the consideration of the anatomical repair of the CMC joint in terms of both bony and ligamentous structures in cases where instability remains after fracture fixation.

## 1. Introduction

Isolated fractures of the trapezium are rare injuries that account for 3–5% of all carpal fractures [1]. Pure carpometacarpal (CMC) dislocations of the thumb are also rare, accounting for less than 1% of all hand injuries [2]. Herein, we report 3 cases of isolated trapezium fracture with thumb CMC joint dislocation. Good short-term results were obtained; however, long-term complications must be noted. Optimal treatment for the injury considering the anatomical features of the thumb CMC joint is discussed.

## 2. Case Presentations

### 2.1. Case 1

**Figure 1:**
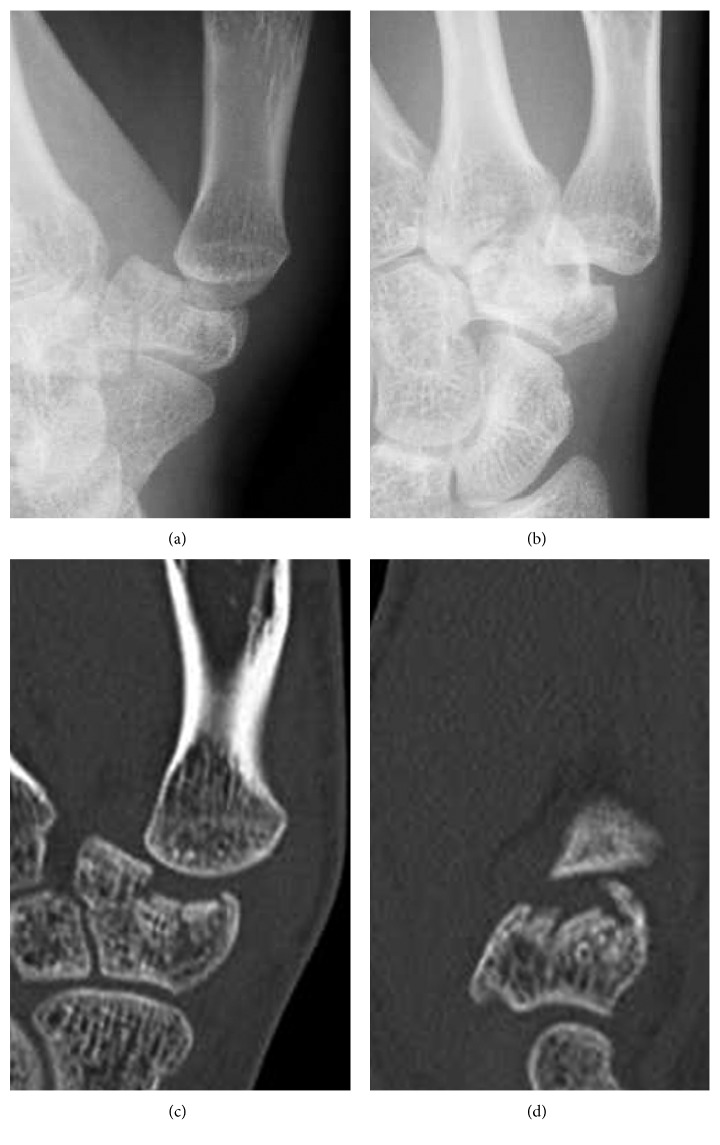
(a, b) Radiographs taken at the initial presentation. The trapezium body fracture and radial dislocation of the CMC joint are shown. (c, d) Computed tomography taken at the initial presentation. The collapse of the trapezium articular surface is shown. (a, c: anteroposterior view; b, d: lateral view.)

**Figure 2:**
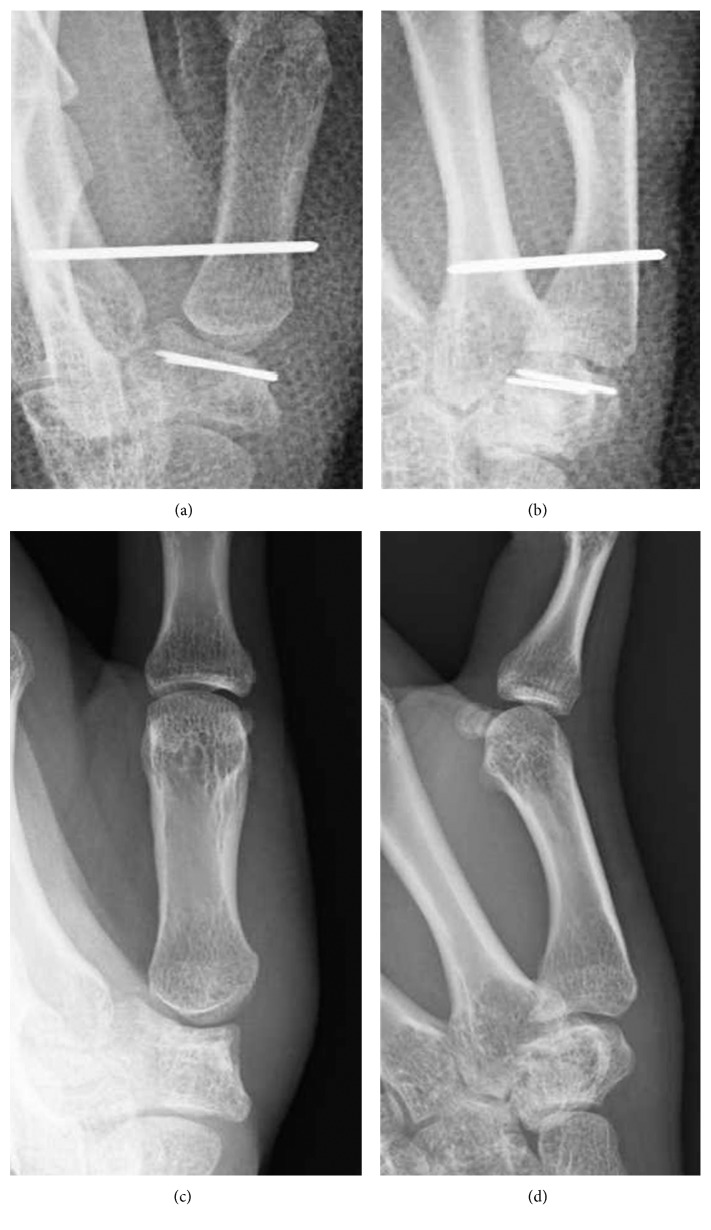
(a, b) Radiographs taken directly after surgery. The collapsed articular surface was elevated, and *β*-TCP was used to fill the subchondral bone loss, fixed by K-wire. (c, d) Radiographs taken at final follow-up, 2 years after surgery. A congruent CMC joint and trapezium fracture union is shown; however, a hyperextension of the thumb metacarpophalangeal (MP) joint was revealed. (a, c: anteroposterior view; b, d: lateral view.)

A 20-year-old female presented to our hospital complaining of pain in her left thumb 2 days after a karate tournament. She had severe pain shortly after she guarded her face with her left forearm, and the opponent's kick directly hit the basal part of her left thumb. On physical examination, there was diffuse swelling and tenderness over her left thumb CMC joint. The range of motion (ROM) of her left thumb was limited due to strong pain. The plain radiograph revealed a trapezium body fracture and radial dislocation of the CMC joint (Figures 1(a) and 1(b)). Computed tomography (CT) showed collapse of the trapezium articular surface (Figures 1(c) and 1(d)). Open reduction and fixation using Kirschner wire (K-wire) was performed 4 days after the initial injury. A radial approach was taken, and the radial joint capsule, along with the radial collateral ligament, was not torn. We incised the capsule and exposed the fracture. The collapsed articular surface was elevated, and *β*-tricalcium phosphate (*β*-TCP) was used to fill the subchondral bone loss, fixed by K-wire (Figures 2(a) and 2(b)). Good stability was obtained by anatomical reduction of the fracture; therefore, we did not examine for a ligament injury. The first and second metacarpals were temporarily fixed by another K-wire. After 6 weeks of thumb spica immobilization, the intermetacarpal K-wire was removed, and active ROM exercises were started. Three months after the initial injury, she had returned to participating in karate. At the final follow-up (2 years after the initial injury), the patient had full ROM in the CMC joint, without pain or instability. Plain radiograph showed a congruent CMC joint and trapezium fracture union. However, a hyperextension of the thumb metacarpophalangeal (MP) joint, a zigzag deformity, was revealed (Figures 2(c) and 2(d)), which is often seen in thumb CMC joint osteoarthritis.

### 2.2. Case 2

**Figure 3:**
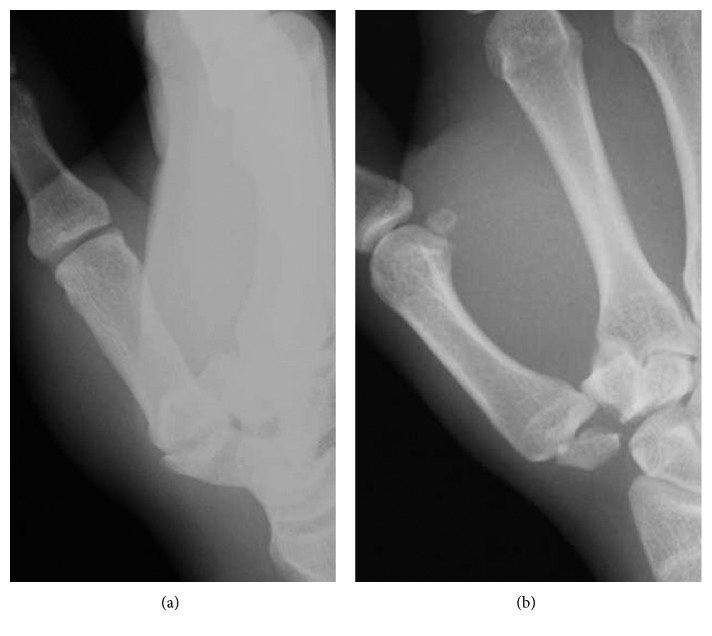
(a, b) Radiographs taken at the initial presentation. The trapezium body fracture and dorsal dislocation of the CMC joint is shown. (a: anteroposterior view; b: lateral view.)

**Figure 4:**
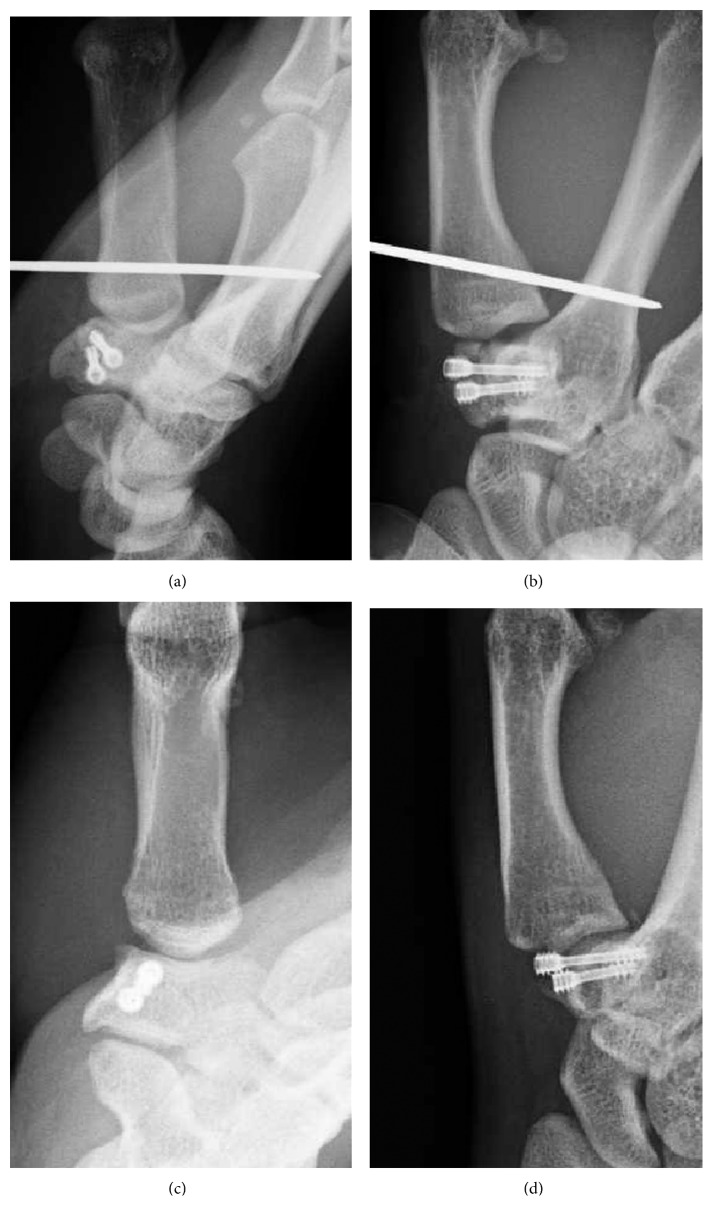
(a, b) Radiographs taken directly after surgery. Good reduction and fixation were obtained using 2 double-thread headless screws. (c, d) Radiographs taken at final follow-up, a year after surgery. A congruent CMC joint and trapezium fracture union is shown. (a, c: anteroposterior view; b, d: lateral view.)

A 17-year-old male presented to our hospital complaining of pain in his left thumb 2 days after a rugby match. The injury involved a rugby-tackling accident; however, the details were not clear. On physical examination, there was diffuse swelling and tenderness over his left thumb CMC joint. The ROM of his left thumb was limited due to strong pain. The plain radiograph revealed a trapezium body fracture and dorsal dislocation of the CMC joint (Figures 3(a) and 3(b)). Open reduction and internal fixation using double-thread headless screws (DTJ screw, Meira^©^, Nagoya, Japan) was performed 5 days after the initial injury. A dorsal approach was taken, and the dorsal joint capsule was not torn. Good reduction and fixation was achieved using 2 DTJ screws. Good stability was obtained by anatomical reduction of the fracture; therefore, we did not examine for a ligament injury. The first and second metacarpals were temporarily fixed by K-wire (Figures 4(a) and 4(b)). After 4 weeks of thumb spica immobilization, the intermetacarpal K-wire was removed, and ROM exercises were started. Three months after the initial injury, he had returned to playing rugby. At the final follow-up (one year after the initial injury), the patient had full ROM in the CMC joint, without pain or instability. Plain radiograph showed a congruent CMC joint and trapezium fracture union (Figures 4(c) and 4(d)).

### 2.3. Case 3

**Figure 5:**
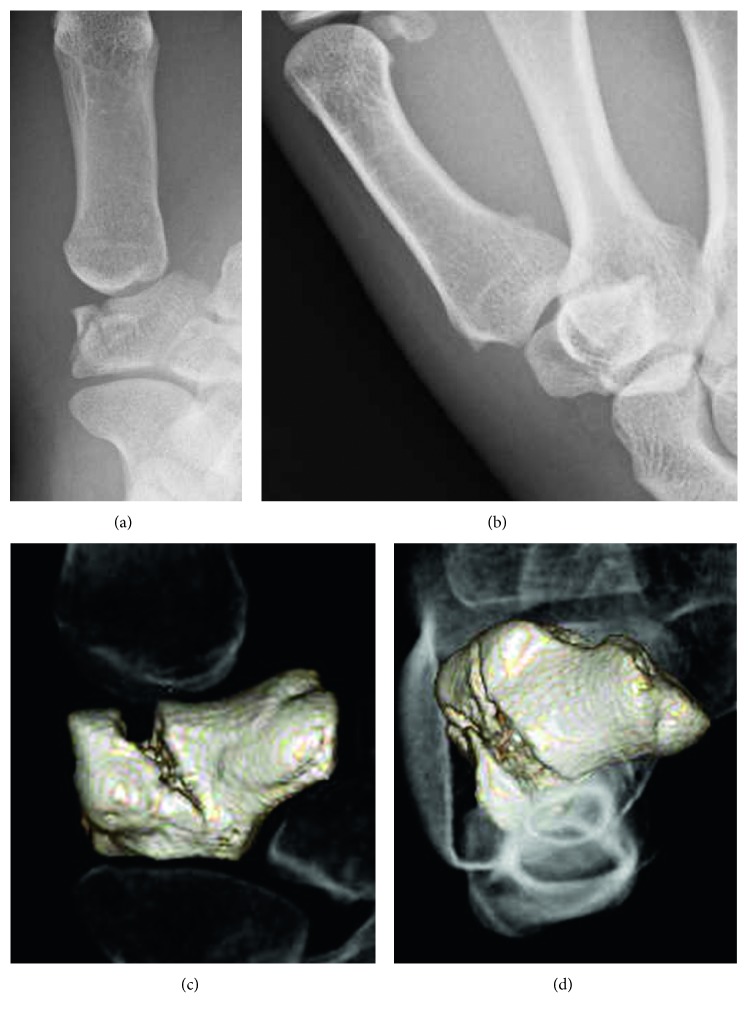
(a, b) Radiographs taken at the initial presentation. The trapezium body fracture and dorsal dislocation of the CMC joint is shown. (c, d) Reconstructed 3D computed tomography taken at the initial presentation. The split in the trapezium body and articular surface is shown. (a, c: anteroposterior view; b, d: lateral view.)

**Figure 6:**
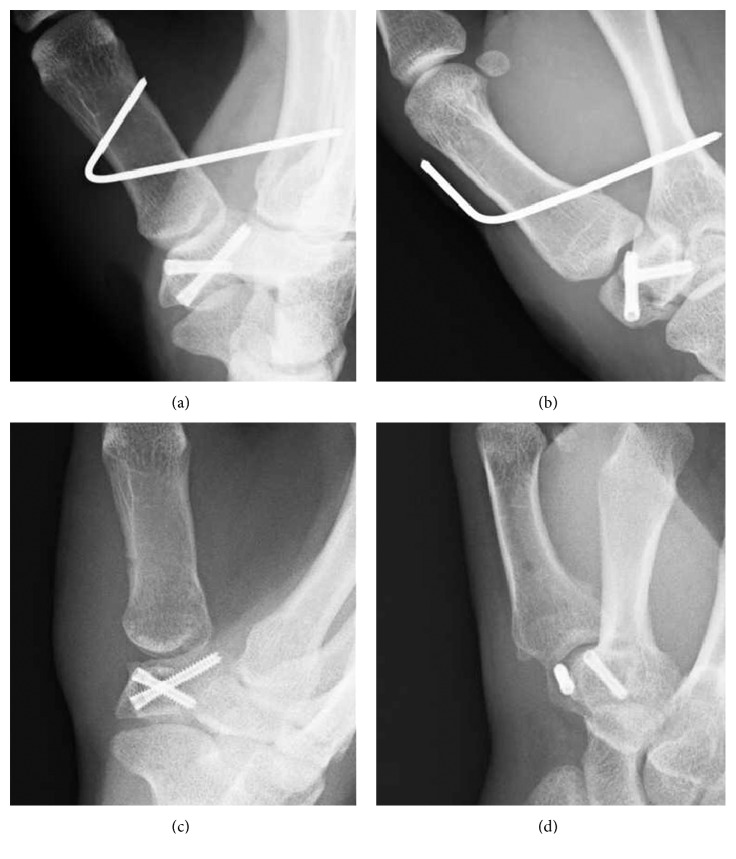
(a, b) Radiographs taken directly after surgery. Good reduction and fixation were obtained using 2-headless screws. (c, d) Radiographs taken at final follow-up, 6 months after surgery. A congruent CMC joint and trapezium fracture union had been obtained. (a, c: anteroposterior view; b, d: lateral view.)

A 17-year-old male presented to our hospital complaining of pain in his right thumb 2 days after a rugby match. He had tackled an opponent and fell down with his right hand grabbing the opponent's jersey. On physical examination, there was diffuse swelling and tenderness over his right CMC joint. The ROM of his right thumb was limited due to strong pain. The plain radiograph revealed a trapezium body fracture and dorsal dislocation of the CMC joint (Figures 5(a) and 5(b)). CT revealed a split fracture of the trapezium body (Figures 5(c) and 5(d)). Seven days after the initial injury, the fracture was percutaneously fixed with 2 headless screws (Acutrak 2 micro, Acumed^©^, Oregon, US). Good reduction was obtained by traction of the thumb and compression of the trapezium from the dorsal aspect. After screw fixation, the CMC joint was highly stable. The first and second metacarpals were temporarily fixed by K-wire (Figures 6(a) and 6(b)). After 4 weeks of thumb spica immobilization, the intermetacarpal K-wire was removed, and ROM exercises were started. Three months after the initial injury, he had returned to playing rugby. At the final follow-up (6 months after the initial injury), the patient had full ROM in the CMC joint, without pain or instability. Plain radiograph showed a congruent CMC joint and trapezium fracture union (Figures 6(c) and 6(d)).

## 3. Discussion

**Figure 7:**
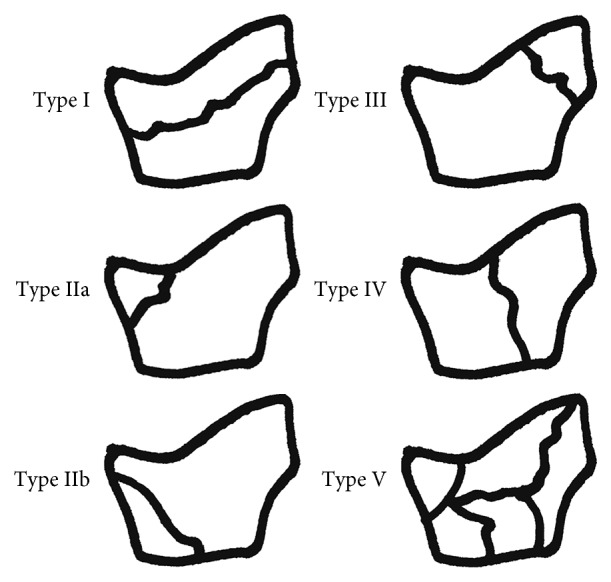
Walker classification (as specified in [16]).

Given that isolated trapezium fracture and thumb CMC joint dislocation are rare injuries [1, 2], the combination is extremely rare, with only 14 cases reported in the English literature [3–15]. Walker et al. [16] classified trapezium fractures into five patterns (Figure 7). In previously reported cases, fracture patterns were either type IIa or IV. In the current study, the fracture pattern of case 1 was type V, and the fracture patterns of cases 2 and 3 were type IV. The mechanism underlying thumb CMC dislocation associated with isolated trapezium fracture is still controversial. Ramoutar et al. [3] indicated a direct dorsoradial impaction or indirect axial loading as mechanisms for the injury, which is consistent with case 1 in the current study. Kose et al. [4] reported either axial loading on a flexed thumb or commissural shearing forces acting on the first web space as the mechanism of injury, most likely consistent with the mechanism of the injury for cases 2 and 3 in the current study.

No consensus exists regarding optimal treatment for this rare injury. In previously reported cases, conservative therapy [4, 5], closed reduction with K-wire fixation [3, 6, 7], open reduction with K-wire fixation [8–10], open reduction with internal fixation [11–13, 15], and closed reduction with external fixation [14] were performed, and excellent results have been reported (Table 1). Bosmans et al. [2] stipulated that treatment depends on the degree of instability and anatomic restoration after the first reduction.

Anatomically, the thumb CMC joint, with its unique articular shape, is stabilized by surrounding ligaments. The biconcave surfaces of the thumb CMC joint allow for a high degree of mobility but, on the other hand, are very unstable [17]. Su et al. reported in their literature that thumb CMC joint would show greater joint gliding in the ulnar-radial direction during thumb abduction and adduction, while greater joint gliding occurs in the dorsal-volar and distal-proximal directions during thumb flexion and extension [18]. The ligaments providing stability to the joint are the anterior oblique ligament (AOL), intermetacarpal ligament (IML), dorsoradial ligament (DRL), posterior oblique ligament (POL), and ulnar collateral ligament (UCL) [19, 20]. Historically, the AOL was considered to be the primary stabilizer of the thumb [21]. Bettinger et al. reported that the DRL and deep AOL play a substantial role in stabilizing the thumb CMC joint [20]. Recently, D'Agostino et al. concluded in their literature that DRL is the strongest and stiffest ligament of the thumb CMC joint [22]. Considering these anatomical studies, when the thumb CMC joint dislocates, the condition of the DRL must be carefully evaluated.

McGuigan and Culp [23] reported that 5 out of 11 patients with an intra-articular trapezium fracture who underwent surgical treatment showed degenerative changes at long-term follow-up (mean, 47 months). Although thumb CMC dislocation was not included in their study, this result suggests that potential ligamentous injuries accompanied the intra-articular fracture, which led to substantial instability of the thumb CMC joint, resulting in osteoarthritis of the joint combined with articular cartilage injury. Although the fracture was anatomically reduced and the thumb CMC joint showed good stability clinically in case 1 of the current study, the radiographic finding at the 2-year follow-up showed a zigzag deformity of the joint. The articular cartilage injury combined with potential malfunctioning of DRL (primary stabilizer of the joint) may have led to this result. Although ligament reconstruction is currently thought to be too aggressive as the primary treatment for this injury [2], there is no consensus regarding optimal treatment. In the previously reported cases, Mody and Dias [8] and Roger et al. [15] treated their cases with ligament reconstruction in addition to K-wire or internal fixation, due to obvious intraoperative instability of the CMC joint. In order to avoid long-term complications such as those seen as in the current case 1, we must strictly assess the instability of the joint. In patients with any degree of instability after fracture fixation, we propose the consideration of open repair of the ligament for better stabilization of the thumb CMC joint. The articular cartilage injury alone may lead to osteoarthritis of the joint during long-term follow-up, and this must be thoroughly explained to patients with trapezium fracture and thumb CMC joint dislocation as well as with other joint intra-articular fractures.

## 4. Conclusion

We have successfully treated 3 cases of isolated trapezium fracture with thumb CMC joint dislocation. Similar to the current cases, good short-term results have been previously reported. However, in order to obtain an excellent long-term clinical result, we must consider the anatomical repair of the CMC joint in terms of ligamentous structures in cases where instability remains after fracture fixation.

## Figures and Tables

**Table 1:** 

Case report	Sex	Age (year)	Mechanism of injury	Trapezium fracture classification	Treatment	Follow-up (months)	Result
Ramoutar et al. [3]	M	27	Football (fell onto out-stretched hand)	IIa	Closed reduction and K-wire fixation	6	Excellent
Kose et al. [4]	M	32	Motorbike accident	IIa	Closed reduction and splinting for 6 weeks	6	Excellent
Tolat and Jones [5]	M	14	Skateboard (fell onto out-stretched hand)	IIa	Closed reduction and splinting for 6 weeks	2	Excellent
Kukreti and Harrington [6]	NA	26	Rugby	IIa	Closed reduction and K-wire fixation	12	Slight pain, minimal loss of CMC flexion
Afshar and Mirzatoloei[7]	M	30	Motorbike accident	IIa	Closed reduction and K-wire fixation	NA	Excellent
Mody and Dias [8]	M	24	Motorbike accident	IIa	Open reduction and K-wire fixation, ligament reconstruction	6	Excellent
Chamseddine et al. [9]	M	23	Road accident	IV	Open reduction and K-wire fixation	6	Excellent
Mumtaz and Drabu [10]	M	14	Hammer hit	IV	K-wire fixation	12	Gross impairment in opposition and abduction
Garavaglia et al. [11]	F	20	Fell while holding the handle of a bucket	IIa	Open reduction and screw fixation	12	Excellent
Garneti and Tuson [12]	M	24	Rugby	IV	Open reduction and internal fixation	12	Excellent
	M	18	Rugby	IV	Open reduction and internal fixation	9	Excellent
Morizaki and Miura [13]	M	31	Fell onto flexed thumb	IIa	Open reduction and internal fixation	12	Excellent
Parker et al. [14]	M	12	Rollerblade (fell onto out-stretched hand)	IIa	Closed reduction and external fixation	36	Excellent
Roger et al. [15]	M	25	Rugby	IV	Open reduction, internal fixation and ligament reconstruction	16	Excellent, but reduced pinch strength
Current case 1	F	20	Karate	IV	Open reduction and internal fixation	24	Excellent, but with a zigzag deformity
Current case 2	M	17	Rugby	IV	Open reduction and internal fixation	12	Excellent
Current case 3	M	17	Rugby	IV	Closed reduction and internal fixation	6	Excellent

CMC: carpometacarpal; NA: not available.
